# Genetic characterization of human echinococcosis in Southern Punjab, Pakistan

**DOI:** 10.3389/fcimb.2023.1141192

**Published:** 2023-04-27

**Authors:** Nosheen Basharat, Jadoon Khan, Irfan Ullah, Aamer Ali Shah, Ijaz Ali

**Affiliations:** ^1^ Department of Biosciences, COMSATS University Islamabad, Islamabad, Pakistan; ^2^ Department of Microbiology, Faculty of Biological Sciences, Quaid-i-Azam University, Islamabad, Pakistan; ^3^ Department of Rehabilitation and Health Sciences, Iqra University (Chak Shahzad), Islamabad, Pakistan; ^4^ Center for Applied Mathematics and Bioinformatics (CAMB), Gulf University for Science and Technology, West Mishref, Kuwait

**Keywords:** human echinococcosis, Echinococcus granulosus, Echinococcus multilocularis, Echinococcus canadensis, mtDNA

## Abstract

**Introduction:**

Echinococcosis is a neglected tropical zoonotic infection that affects both the human and livestock populations. In Pakistan, the infection is long-standing, but data on its molecular epidemiology and genotypic characterization in the southern Punjab region are limited. The aim of the current study was the molecular characterization of human echinococcosis in southern Punjab, Pakistan.

**Methods:**

Echinococcal cysts were obtained from a total of 28 surgically treated patients. Patients’ demographic characteristics were also recorded. The cyst samples were subjected to further processing to isolate DNA in order to probe the *Nad1* and *Cyt-b* genes, followed by DNA sequencing and phylogenetic analysis for genotypic identification.

**Results:**

The majority of the echinococcal cysts were from male patients (60.7%). The liver was the most commonly infected organ (60.71%), followed by the lungs (25%), spleen (7.14%), and the mesentery (7.14%). Molecular and genotypic identification through sequencing and phylogenetic tree analysis showed that most of the cysts (24/28, 85.7%) were caused by the species *Echinococcus granulosus sensu stricto* (*E. granulosus s.s.*) (G1 and G3), followed by *Echinococcus multilocularis* (*E. multilocularis*) and *Echinococcus canadensis* (*E. canadensis*) (G6/G7) (3/28, 10.8%, and 1/28, 3.5%, respectively).

**Conclusion:**

The current study concluded that the majority of human infections were caused by *E. granulosus s.s.*, followed by the *E. multilocularis* and *E. canadensis* species (G6/G7). Genotypic characterization among both human and livestock populations is needed to explore the genetic diversity of echinococcosis.

## 1 Introduction

Echinococcosis is a zoonotic disease that affects approximately 2–3 million people worldwide (Agudelo et al., 2016). The World Health Organization (WHO) has included echinococcosis in its list of 17 neglected tropical diseases (NTDs) (Khan et al., 2018). The disease accounts for one million disability-adjusted life years (DALYs) per annum, and the cost needed to address it, including treatment for affected individuals and livestock losses, is approximately $3 billion per year (Agudelo et al., 2016). Echinococcosis is caused by various species of the helminth parasite *Echinococcus granulosus sensu lato* (*E. granulosus s.l.*), which infects both humans and mammals. According to the current international consensus, the *E. granulosus s.l.* cluster comprises nine species: *E. granulosus* (Gill and Rao, 1967) *sensu stricto* (G1–G3), *E. canadensis* (G6–G10) (Pavia et al., 2020), *E. ortleppi* (G5) (LopezNeyra and Soler Planas, 1943), *E. felidis* (Ortlepp, 1937), and *E. equinus* (G4) (Williams and Sweatman, 1963). Human echinococcosis takes the form of either cystic echinococcosis (CE), caused by *E. granulosus*, or alveolar echinococcosis (AE), caused by *E. multilocularis* (Eckert and Deplazes, 2004).

To complete their life cycle, *Echinococcus* spp. need two different hosts. The intermediate host (mainly herbivores or humans) is infected by ingesting the eggs generated in the small intestine of the definitive host (dogs and other canids). Humans are considered accidental (dead-end) hosts. CE is generally asymptomatic until the cyst becomes fully developed and exerts pressure on neighboring tissues/organs, i.e., the biliary tract, liver, bronchus, heart, pancreas, spleen, or brain (Salem et al., 2011; Agudelo et al., 2016). Infection is highly prevalent in areas of the world where the population depends on livestock, and where health facilities are poor and socioeconomic and literacy levels are low (Craig et al., 2017). Humans can also become infected by ingesting contaminated fruit and vegetables that have not been properly washed or water from contaminated sources, or through contact with the fur of infected dogs (Robertson et al., 2016).

Echinococcosis seems to be emerging and reemerging in human populations in several geographical locations, such as Asia, the Americas, Europe, and Africa, (Khan et al., 2018): it is endemic in Chile, Central Asia, Peru, western China, Argentina, the Mediterranean region, Uruguay, East Africa, and southern Brazil (Khan et al., 2018). Globally, 95% of human echinococcosis infections are due to *E. granulosus s.s.*, and the disease has been reported in people of all ages, from 1 to 75 years (Fotiou et al., 2012). In Pakistan, echinococcosis was first reported before the Partition of India (Sami, 1938); however, over the last 72 years there have been only a few confirmed cases of the disease in the animal and human populations of the Punjab, Sindh, and Khyber Pakhtunkhwa provinces (Ahmed et al., 2017; Basharat et al., 2021; Khan et al., 2021). Most of the published studies on human echinococcosis in Pakistan are case studies (Jamal and Jafarey, 1989; Biyabani et al., 2000; Ali et al., 2009; Amin et al., 2009; Fatimi et al., 2010; Masroor et al., 2010; Shamim, 2010; Sultana et al., 2012; Nadeem et al., 2013; Khan et al., 2015); only two reports on the genetic characterization of the parasite in humans are available (Ali et al., 2015; Khan et al., 2015). The *Echinococcus* genotypes reported in the human populations of Khyber Pakhtunkhwa and the northern regions of Punjab so far are *E. granulosus s.s.* (G1 and G3) and *E. canadensis* (G6/G7) (Ali et al., 2015; Khan et al., 2020).

However, the present-day disease burden in southern Punjab, Pakistan, is largely unknown. This study was therefore designed to determine the burden of CE and AE among the human population in the southern Punjab region of Pakistan, to determine if it is in a reemerging or controlled state, and to identify the associated etiological agents.

## 2 Materials and methods

### 2.1 Geography of the study area

**Figure 1:**
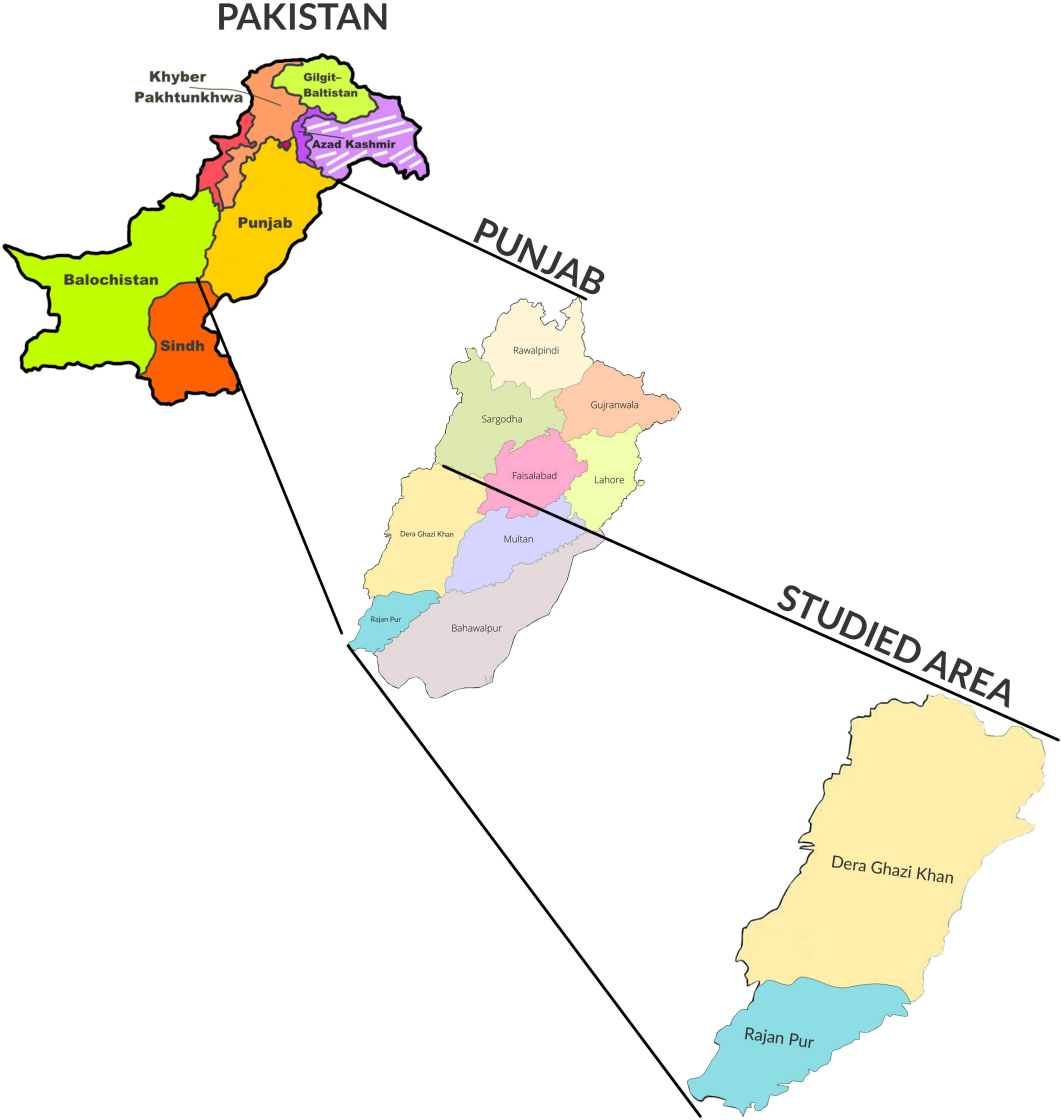
Map of Pakistan showing Punjab province and the selected study area of southern Punjab (specifically, the Dera Ghazi Khan and Rajanpur regions).

Pakistan is a developing country consisting of five provinces, including Punjab, a region that is densely populated, with 110,012,442 residents, according to the 2017 census. It is situated at the center of the country and is bordered by the other provinces, namely Khyber Pakhtunkhwa, Sindh and Balochistan, the Islamabad Capital Territory, and Azad Kashmir. The study areas of Rajanpur and Dera Ghazi (DG) Khan are located in southern Punjab, with latitudes and longitudes of 29°12′28.74″N and 70°14′27″E, and 0°1′56.9496″N and 70°38′24.8784″E, respectively. Both of these areas are regarded as agricultural owing to their hot climates in the summer months and the various breeds of livestock that are reared within them (Khan et al., 2020) (see **Figure 1**).

### 2.2 Study setting

The current study was conducted from September 2018 to September 2019 in the Rajanpur and DG Khan districts of southern Punjab, Pakistan. Experimental work was performed at the Molecular Virology Laboratory, COMSATS University Islamabad (CUI), Islamabad, Pakistan. All individuals whose cysts were included were briefed about the study. Written informed consent was obtained from all individuals and consultants.

### 2.3 Sample collection and processing

A total of 28 echinococcosis cysts were collected after radial surgery and fixed in 10% formalin. All cysts were observed macroscopically, then embedded in paraffin solution in accordance with routine histological procedures. Ten-micrometer cyst sections were stained with hematoxylin and eosin and then examined by light microscopy.

### 2.4 Nucleic acid extraction

Total nucleic acid was extracted from the 28 cyst samples collected during surgery. Tissue samples from the cyst germinal layer were separated and then prepared for genomic extraction using a DNeasy Blood and Tissue Kit (Qiagen, Hilden, Germany), in accordance with the manufacturer’s guidelines. A NanoDrop spectrophotometer (Thermo Fisher Scientific, Wilmington, DE, USA) was used to determine the concentration of the extracted DNA, which was then then stored at –20°C until used for PCR amplification.

### 2.5 PCR amplification

For the identification of *E. granulosus* and *E. multilocularis*, a PCR assay was performed for mitochondrial NADH dehydrogenase I (*Nad1*) and the cytochrome *b* (*Cyt-b*) gene fragment, in accordance with the process described in previous studies (Bowles and McManus, 1993; Wu et al., 2017), with slight modifications to the thermocycler conditions. The PCR conditions were as follows: initial denaturation at 94°C for 5 min, followed by 35 cycles of denaturation at 94°C for 30 s, primer annealing for 45 sec (at 55°C for *E. granulosus* and at 52.5°C in the case of *E. multilocularis*), then initial extension at 72°C for 60 s and a final extension at 72°C for 10 min. The already identified *E. granulosus s.s.* (G1 and G3) and *E. multilocularis* samples provided by the Molecular Virology Laboratory of CUI were used as positive controls. Agarose gel (2%) electrophoresis was used for the separation of the PCR-amplified products; these were then pre-stained with ethidium bromide prior to visualization by UV transillumination according to their fragment size.

### 2.6 Sequence analysis and phylogenetic characterization

The sequencing of PCR products in both directions using a set of forward and reverse primers was carried out by sending to Macrogen (Macrogen Inc., Seoul, South Korea). Sequences were read using Chromas software (Technelysium Pty Ltd., Queensland, Australia); subsequently, the alignment and assembly of both forward and reverse sequences were carried out using DNASIS MAX software (version 3.0; Hitachi, Yokohama, Japan). The random nucleotide BLAST program (http://blast.ncbi.nlm.nih.gov) was used to search for sequences with phylogenetic relatedness in the GenBank database (Altschul et al., 1997). The identified sequences were deposited in GenBank under the submission ID SUB1002748 and bioproject ID PRJNA747636. The comparative alignment of the final sequences of each sample with reference sequences followed by the construction of a phylogenetic tree was carried out using a maximum likelihood method (Taimura–Nei method) with a bootstrap value of 1,000 for the genotypic determination of *Echinococcus* using MEGA X software (www.megasoftware.net) in accordance with the method set out by Kumar et al. (Kumar et al., 2018).

### 2.7 Statistical analysis

All data were fed into SPSS (Statistical Package for the Social Sciences) (version 16.0), and a chi-squared test was used to determine the correlation (with 95% confidence interval) among CE and various factors, such as infected organ and patient’s age and sex. A *p-*value of < 0.05 was considered significant.

## 3 Result

### 3.1 Demographic characterization of the patients

A total of 28 surgically removed cysts (24 from patients in Rajanpur and four from patients in DG Khan) were examined (ratio of male to female patients: 60.7% to 39.3%). Patients were divided into three groups according to age, i.e., age groups 1 (15–30 years), 2 (31–45 years), and 3 (45–70 years). Age and sex were non-significantly correlated with incidence. Among the various diagnostic procedures used, computed tomography (CT) (*p* < 0.05), used to screen infected organs such as the lungs, spleen, and mesentery (*p* < 0.05), was significantly linked with the detection of echinococcal cysts (shown in **Table 1**).

**Table 1:** Incidence rate of echinococcosis in the human population of southern Punjab.

Characteristic	Infected/total	*P*-value	Chi-squared (*χ^2^*)
Sex			
Male	17/28	0.464	1.286
Female	11/28		
Age group			
Age group 1	7/28	0.357	2.214
Age group 2	8/28		
Age group 3	13/28		
Organ distribution			
Liver	17/28	0.464	1.286
Lungs	7/28	0.036	7.000
Spleen	2/28	0.000	20.571
Mesentery	2/28	0.000	20.571
Diagnostic method			
Ultrasound	18/28	0.214	2.286
CT	10/28	0.036	2.286
ELISA	20/28	0.214	5.143

CT, computed tomography; ELISA, enzyme-linked immunosorbent assay.

### 3.2 Molecular identification of the echinococcus

**Figure 2:**
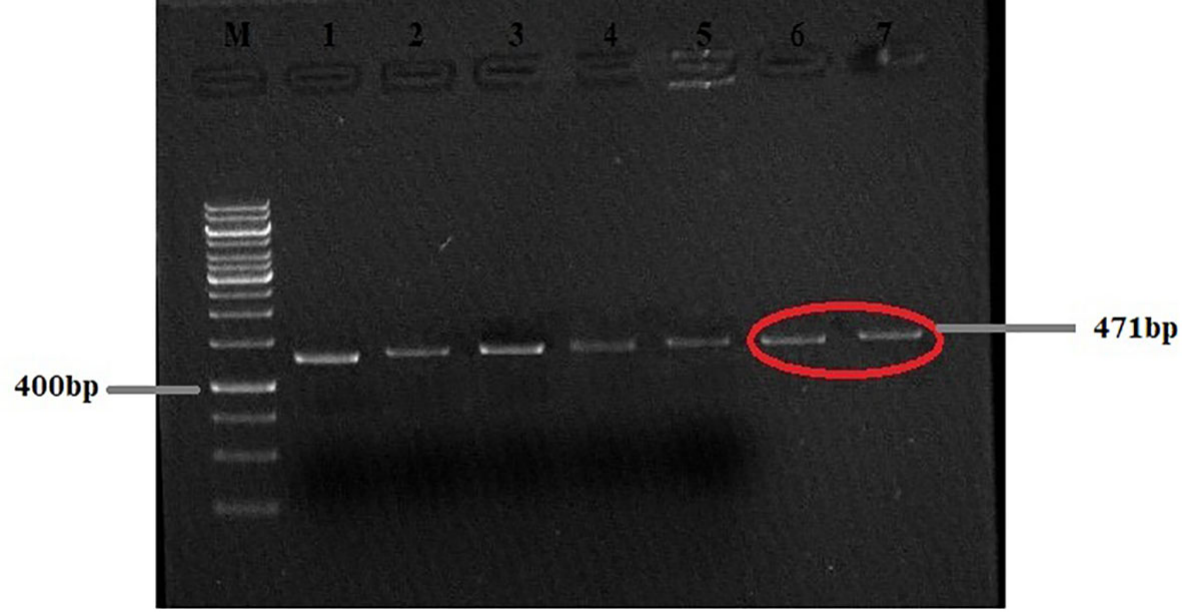
Agarose gel (2%) of *Echinococcus granulosus Nad1* gene PCR-amplified product. M, 50-bp molecular ladder; HC, human cyst (the gels are cropped).

**Figure 3:**
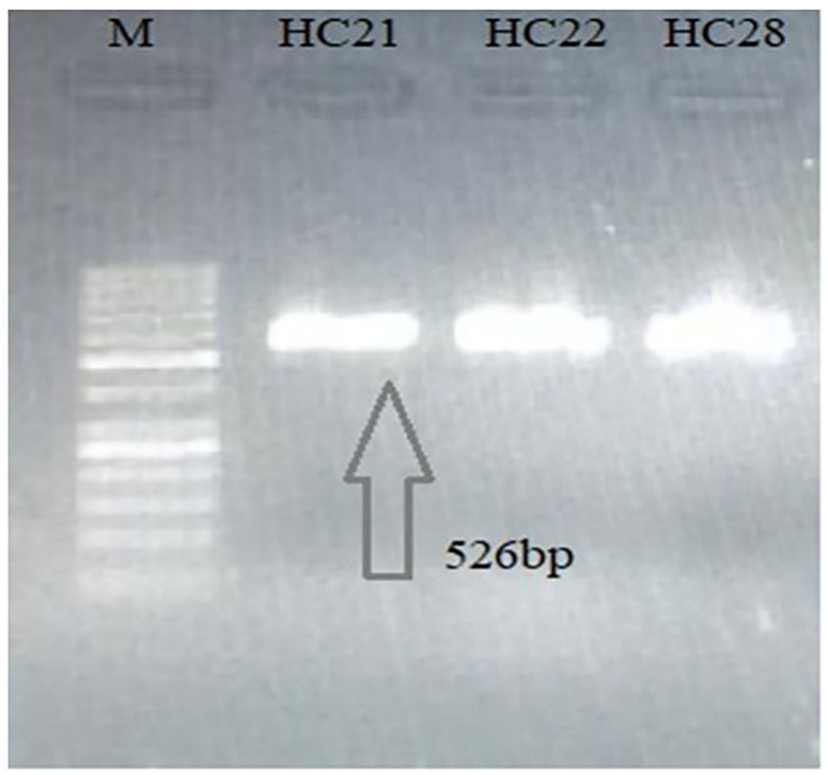
Agarose gel (2%) of *Echinococcus multilocularis Cyt-b* gene PCR-amplified product. M, 50-bp molecular ladder; H, human cyst (the gels are cropped).

PCR-based amplification of the mitochondrial *Nad1* and *Cyt-b* genes revealed that 25 out of the 28 cyst samples (17 from the liver, four from the lungs, two from the spleen, and two from the mesentery) were positive for *E. granulosus s.s*., and that 3 out of the 28 lungs samples were amplified for *E. multilocularis* (**Figures 2**, **3**). A Thermo Fischer GeneRuler 50 bp DNA ladder with the catalog number SMO372 was used to carry out PCR-based amplification.

### 3.3 Genotypic characterization of *Echinococcus* species

**Figure 4:**
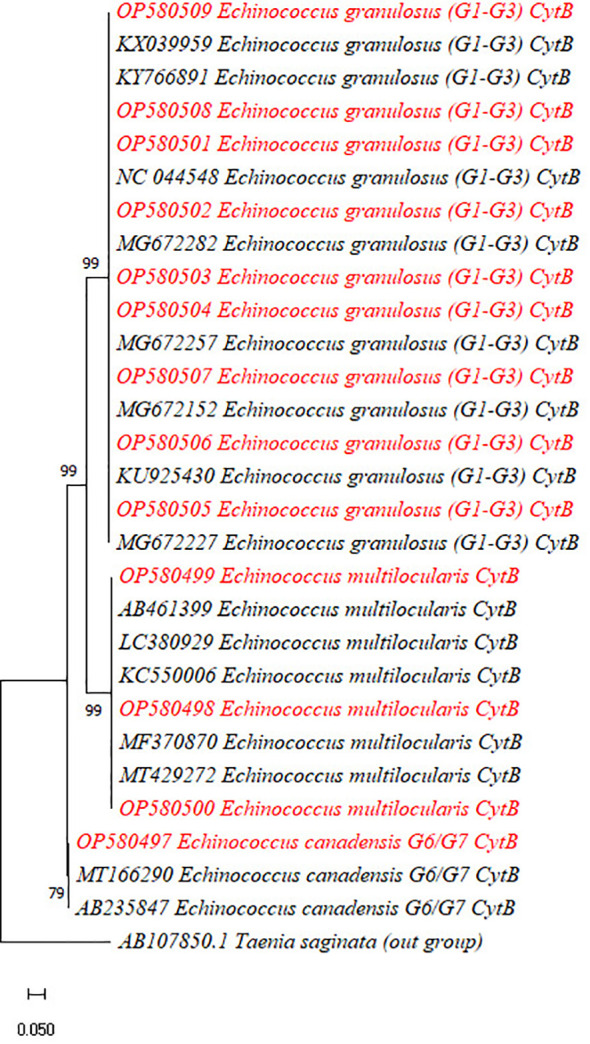
Phylogenetic analysis of the currently identified *Echinococcus granulosus*, *Echinococcus multilocularis*, and *Echinococcus canadensis* species based on the *Cyt-b* gene. The phylogenetic tree was constructed in MEGA X software using maximum likelihood (Taimura–Nei) methods with a bootstrap value of 1,000.

**Figure 5:**
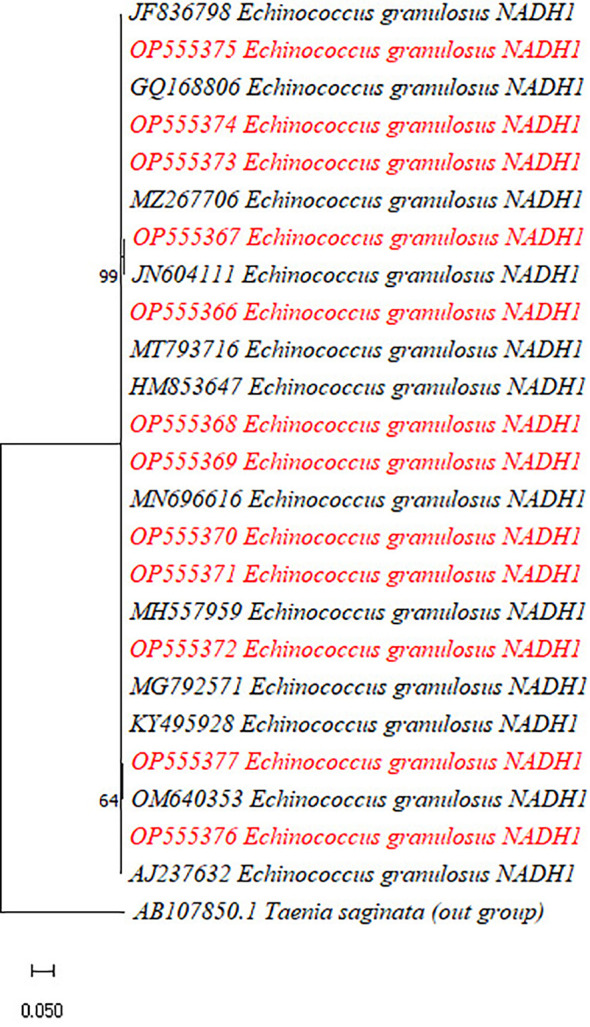
Phylogenetic analysis of the currently identified *Echinococcus granulosus* species based on the *Nad1* gene. The phylogenetic tree was constructed in MEGA X software using maximum likelihood (Taimura–Nei) methods with a bootstrap value of 1,000.

The PCR products of 13 out of 28 *Cyt-b* genes (10 for *E. granulosus* and three for *E. multilocularis*) and 15 out of 28 *Nad1* genes revealed that the majority of the cysts were caused by *E. granulosus s.s.* (G1–G3) (24 out of 28, 85.7%), followed by *E. multilocularis* (i.e., 3 out of 28, 10.5%) and *E. canadensis* (G6/G7) (1 out of 28, 3.5%) (**Figures 4**, **5**).

## 4 Discussion

CE is endemic to the Central Asia, with about 270 million people (58% of the total human population) in western China, Iran, Uzbekistan, Afghanistan, Tajikistan, Mongolia, Kazakhstan, Kyrgyzstan, Turkmenistan, and Pakistan being at risk of contracting this disease (Zhang et al., 2015). However, there is a large gap in the reported data relating to the epidemiological status, incidence rate, actual prevalence, and genotypic characterization of human echinococcosis in Pakistan. Echinococcal cysts are frequently detected in various organs, such as the liver, lungs, spleen, mesentery, brain, and heart (Cerda et al., 2018). Cerda et al. (2018) observed that the liver was the most frequently affected organ, followed by the lungs, spleen, and the mesentery, coinciding with other reports from Pakistan (Butt and Khan, 2019; Khan et al., 2019) and elsewhere (Kamali et al., 2018; Mahmoudi et al., 2019). However, Salem et al. (2011) reported contradictory results, namely that the largest number of echinococcal cysts was detected in the lungs, followed by the liver and other organs (Salem et al., 2011). The reason for the finding of large numbers of cysts in the liver might be that, on initial infection, *Echinococcus* oncosphere larvae travel to the liver through the hepatic portal vein, which makes it more likely that cysts will develop on the liver rather than on other organs (Thompson et al., 2017). In the present study, the largest numbers of echinococcal cysts were detected in age group 3, followed by age groups 2 and 1. Similar reports of higher incidence rates in the > 50 years group were observed by Tiaoying et al. (2005) and Kamali et al. (2018) and in the 41–50 years age group by Zhang et al. (2015). Conversely, incidence rates have also been observed to be higher in the 20–29 years and 21–30 years age groups than in older individuals (Khan et al., 2019; Muqaddas et al., 2019). It could therefore be suggested that echinococcosis can occur in any age group; however, because of their chronic and slow-growing nature, the likelihood of cysts developing is directly proportional to age, as reported by Zhang et al. (2015).

Using sequencing technology and phylogenetic tree analysis, this study found that the majority of echinococcal cysts (85.7%) are caused by *E. granulosus s.s.* (G1–G3), corroborating previous reports of the high prevalence of *E. granulosus s.s.* (Pezeshki et al., 2013; Rojas et al., 2014; Ali et al., 2015; Zhang et al., 2015; Kinkar et al., 2017; Nikmanesh et al., 2017; Moradi et al., 2019; Khan et al., 2020). The reason why the G1–G3 genotypes are globally the most prevalent might be the trade in domestic animals (carrying the *E. granulosus s.s.* G1–G3 genotypes) from Iran to India and Italy (Kinkar et al., 2018a) and the spread of the G1 genotype from Türkiye to other countries worldwide (Sharbatkhori et al., 2009; Pezeshki et al., 2013; Rojas et al., 2014; Farhadi et al., 2015; Rostami et al., 2015; Nikmanesh et al., 2017; Spotin et al., 2017; Jafari et al., 2018; Moradi et al., 2019). The widespread presence and continuous circulation of the *E. granulosus s.s.* G1–G3 genotypes might also be due to the presence of a diverse range of intermediate hosts globally (Romig et al., 2015; Romig et al., 2017; Kinkar et al., 2018b), a factor that might also have contributed to the high genetic diversity reported within *E. granulosus s.s.* (Kinkar et al., 2018b).

A recent study has reported that cases in China account for 10.7% of global human echinococcosis infections caused by *E. multilocularis* (Giraudoux et al., 2003); high infection rates have also been reported in the adjacent areas of Punjab (Khan et al., 2020) and Khyber Pakhtunkhwa, both in Pakistan (Ali et al., 2015), as well as in the northern hemisphere, and even in neighboring Afghanistan (Deplazes et al., 2017). Infection with *E. multilocularis* has also been reported in humans in Türkiye, the Netherlands, Poland, and North America (Inceboz et al., 2005; Chauchet et al., 2014; Massolo et al., 2014; Kowalczyk et al., 2019). The aforementioned studies indicate that both *E. granulosus* and *E. multilocularis* are involved in human infection; however, species variation might be due to the difference in their virulence and pathogenicity, non-availability of various species-specific ELISAs, as well as limited data about the genetic characterization of *Echinococcus* spp. in Pakistan and all over the world.

This study found the prevalence of G6 and G7 genotypes to be 3.5% (1 out of 28 patients); these genotypes have previously been detected in both Punjab and Khyber Pakhoonkhwa, Pakistan (Ali et al., 2015; Khan et al., 2020), through PCR–restriction fragment length polymorphism (RFLP). In addition, the Pakistan genotypes, G6 and G7, have also been detected in neighboring countries such as Iran, suggesting that, after the genotypes of the G1–G3 complex, G6 is the second most prevalent genotype causing human infection with cystic echinococcosis (*E. granulosus s.s.).* The low prevalence of the G6 and G7 genotypes in *E. granulosus*-endemic areas indicates that they exert a minor impact on human health (Romig et al., 2015; Moradi et al., 2019), although a higher prevalence of G6 and G7 isolates in human CE infections is reported in countries where livestock infection by *E. granulosus s.s.* is rare (Romig et al., 2015) Studies conducted in Kerman (south-eastern and eastern), Iran, found that infected hosts had a higher prevalence of the *E. granulosus* G6 genotypes than of the G1 genotype (Rostami et al., 2015; Karamian et al., 2017). In Pakistan, the detection and treatment of echinococcosis is currently not prioritized (Khan et al., 2019); however, the present study recommends that CE and AE be regarded as important public health concerns, as they are highly prevalent in rural areas of the country.

The most important limitation of the current study was the determination of *E. granulosus* with the sequencing of only two partial mtDNA genes, i.e., *Nad1* and *Cyt-b*, as this is not sufficient for the complete identification of each genotype within the G1–G3 complex (Kinkar et al., 2017; Kinkar et al., 2018a; Kinkar et al., 2018b). Other barriers included difficulty in obtaining permission to collect echinococcal cysts from hospitalized patients, because of a lack of physician and surgeon interest in our research, and our inability to collect ethanol-embedded cyst tissue, which yield more accurate nucleic acid extractions, sequencing, and phylogenetic analysis results.

## 5 Conclusion

In conclusion, this study confirmed the presence of *Echinococcus* spp., i.e., *E. granulosus s.l., E. multilocularis*, and *E. canadensis* in human echinococcal cysts extracted from patients in southern Punjab. This zoonotic infection represents a potential threat to human populations, and, as such, effective control measures should be designed and implemented to limit the likelihood of future human infection.

## Data availability statement

The dataset presented in this study can be found at https://www.ncbi.nlm.nih.gov/genbank/ (accession number OP580497-OP580509 and OP555366-OP555377).

## Ethics statement

The studies involving human participants were reviewed and approved by Ethical Approval Committee of COMSATS University, Islamabad, under reference no. CUI-Reg/Notif. 2255/19/2661. The patients/participants provided their written informed consent to participate in this study.

## Author contributions

IA and AS were involved in supervision, funding acquisition, review, and editing. NB, JK, and IU developed the methodology, carried out sample collection and experiment, and wrote the original manuscript. All authors contributed to the article and approved the submitted version.
